# The Vista of Application of Specific Anaphylaxis Accurate Diagnosis Based on DNA Single-Nucleotide Methylation Sites

**DOI:** 10.1155/2021/8202068

**Published:** 2021-11-24

**Authors:** Xiangjie Guo, Yaqin Bai, Hualin Guo, Peng Wu, Hao Li, Liqin Zhai, Yan Feng, Jianguo Li, Cairong Gao, Keming Yun

**Affiliations:** ^1^Department of Forensic Medicine, Shanxi Medical University, Taiyuan, ShanXi, China; ^2^Shanxi Key Laboratory of Drug Toxicology and Drug for Radiation Injury, China Institute for Radiation Protection, Taiyuan, ShanXi, China; ^3^Pathologic Department of People's Hospital of Shanxi Province, Taiyuan, Shanxi, China; ^4^Department of Otolaryngology Head & Neck Surgery, The First Hospital Shanxi Medical University, Taiyuan, Shanxi, China

## Abstract

Anaphylaxis has rapidly spread around the world in the last several decades. Environmental factors seem to play a major role, and epigenetic marks, especially DNA methylation, get more attention. We discussed several GEO opening data classifications with TOP 100 specific methylation region values (normalized M-values on line) by machine learning, which are remarkable to classify specific anaphylaxis after monoallergen exposure. Then, we sequenced the whole-genome DNA methylation of six people (3 wormwood monoallergen atopic rhinitis patients and 3 normal-immune people) during the pollen season and analyzed the difference of the single nucleotide and DNA region. The results' divergences were obvious (the differential single nucleotides were mostly distributed in nongene regions but the differential DNA regions of GWAS, on the other hand), which may have caused most single nucleotides to be concealed in the regions' sequences. Therefore, we suggest that we should conduct more “pragmatic” and directly find special single-nucleotide changes after exposure to atopic allergens instead of complex correlativity. It is possible to try to use DNA methylation marks to accurately diagnose anaphylaxis and form a machine learning classification based on the single methylated CpGs.

## 1. Introduction

In the past few decades, the allergic disease incidence rate has increased yearly at a rapid rate, and these changes are much faster than the genome. The incidence of anaphylaxis ranges from 1 to 761 per 100000 person-years for total anaphylaxis and 1 to 77 per 100 000 person-years for food-induced anaphylaxis worldwide [1]. In Taiwan, the incidence rate of anaphylaxis has increased at an average rate of 5% annually since 2001 [2]. Allergic rhinitis is the most common chronic allergic disease, and its incidence is rising in parallel with other IgE-mediated diseases, affecting 10 to 30% of adults and up to 40% of children [3, 4]. In some Western developed countries, food-induced anaphylaxis already seems to be an epidemic (and highest in children) [5, 6]. However, among the Asian population, the incidence of drug-induced anaphylaxis increased faster compared with other types. Also, in some developing countries, such as Brazil, anaphylactic shock has high incidence rate [7]. Different age groups focused on distinct allergic incentives, and the incidence of allergies increased over time [8]. The large-scale intervention trials for food allergy support that the decrease in early exposure to allergens will increase the risk of food allergy [6]. The distinguishable type of allergy incidence varies significantly in different countries, even within countries, suggesting environmental factors play a major role compared with genetic factors in these changes [9]. The rapid development of the global economy, upturn living standards, and lifestyle changes are accelerating the increased number of allergic cases and new allergens, which is a challenge for accurate diagnosis in forensic medicine and the medical domain.

### 1.1. The Relation of Epigenetics and Allergy

The rapid global outbreak of anaphylaxis is inevitable. To date, genome-wide linkage and association studies have identified many allergy-associated genes or loci [10–12]. However, the pattern of genetic susceptibility cannot explain all the risks of anaphylactic raise. There is evidence that the risk of allergy is higher in mothers than in fathers, and it is hereditable [13]. Because epigenetic marks are also heritable and capture responses to environmental factors [14, 15], it is logical that epigenetics plays an important role in the event of allergy. Furthermore, epigenome changes can be altered by many environmental exposures and often lead to rapid and persistent changes in gene expression [16]. Though monozygotic twins with the same genetic background are discordant for allergic rhinitis, they differ in peripheral blood mononuclear cell (PBMC) gene expression levels, and the sensitization of familiar allergens differs because of environmental contributions [17, 18]. In some studies, it has been determined that the influences can modify allergic patients' gene expression through DNA methylation [14, 19–21]. Thus, DNA methylation as an epigenetic mark represents a logical way to reflect allergy disease conditions.

### 1.2. The Association between DNA Methylation and Gene

DNA methylation refers to the covalent bonding of a methyl group to the 5th carbon position of the cytosine of the genomic CpG dinucleotide under the action of DNA methyltransferase. DNA methylation is observed in different sequences; however, it is almost exclusively found in CpG dinucleotides in humans. There are CpG-rich sequences termed CpG islands (CGI), which are generally unmethylated [22] and associated with histone modifications such as H3K4me [23, 24], but dissociative CpGs are methylated in general. There are around 50,000 CGIs in the human genome and more dissociative CpGs [25]. High CpG methylation in genomes increases the frequency of spontaneous mutations because methylated C residues spontaneously deaminate to form T and CpG steadily to TpG, which evidenced that the actually observed numbers of CpG are less than the expected (only around 21%) [25, 26]. It is the potential cement of evolution, which maybe one of the methods by which biological phenotypes by change from environmental factors are inherited.

**Figure 1:**
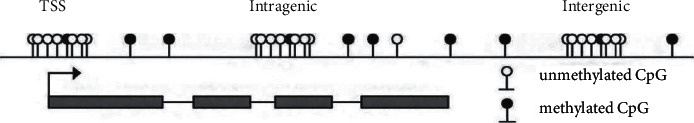
The ubiety between DNA methylation and gene. These include “TSS,” “Intragenic,” “Intergenic,” and CpG alone.

As a DNA molecule's cytosine is methylated, there is a positional correlation between genes and DNA methylations. DNA methylations occur during or outside annotated genes in the genome (Figure 1). It is associated with gene silence that changes the biological phenotype and gene expression to determine the cellular types and functions with CpG methylation. In general, the conserved CGIs on transcription start sites (TSSs) are highly methylated and can influence transcription of genes by impeding the binding of transcriptional proteins. However, CGIs located between genes or transcriptions are observed to be highly tissue specific [27, 28], which are highly methylated and control gene expression patterns to determine the cellular types and functions in the process of cell differentiation by H3K4me3 or some potential methods [29]. The great mass of regions in the whole genome's functions is unclear. In other diseases, the region of DNA methylation change plays a role in the expression of disease-related genes and may become a new TSS. It reminds us that we should pay attention to all DNA methylation sites, not only TSS, when exploring the relation with allergy.

Machine learning is a field of computer science, which gives computer systems the ability to “lean” with data [30, 31]. Machine learning analyses data to study the construction of algorithms, which can make predictions on data, produce reliable, repeatable decisions and results, and uncover some “hidden insights” [32] and handle more complicated and bulky data. Decision tree learning is a method commonly used in data mining that includes classification tree analysis where the predicted outcome is the class to which the data belongs [33].

### 1.3. Idea

Whether DNA methylation such as epigenetics mark can be used to diagnose allergy and how should it be applied? It has been proposed, based on a few specific DNA methylation marks' joint detection, considering that sequencing and microarray at the whole genome are time consuming, costly, and difficult to popularize, to classify anaphylaxis types by randomForest (one of the decision tree learning) of the R programming language (R). Also, it inspires us that some researchers have presented an approach for the DNA methylation-based classification of 100 known central nervous system tumours that is based on machine learning, which can obtain accurate diagnosis and avoid observer errors by using Infinium HumanMethylation450K BeadChip arrays data recently [34]. Then, we plan to use these methods to prove the idea and find the mark types from some selected opening GEO data and the sequencing data.

## 2. Results

### 2.1. GEO Data Analysis Results

**Figure 2:**
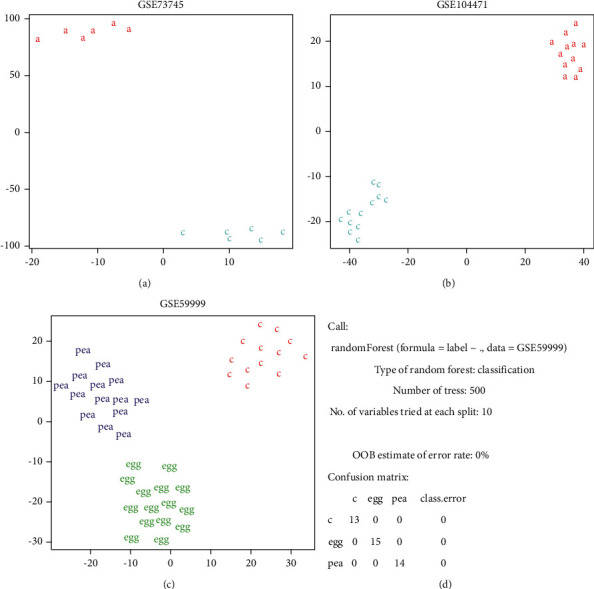
(a) The result of GSE73745 by t-SNE. “a” represents respiratory allergy patients, and “c” represents healthy controls. (b) The result of GSE104471 by t-SNE. “a” represents allergic asthma patients, and “c” represents healthy controls. (c) The result of GSE59999 by t-SNE. “Egg” represents egg-allergic patients, “pea” represents peanut-allergic patients, and “c” represents healthy controls. (d) The result of GSE59999 by randomForest.

We analyzed allergy-associated hematic DNA regions' methylation from several GEO datasets by our methods. Some of the results of GSE73745 [35], GSE104471 [36], and GSE59999 [37] are shown in Figure 2. GSE73745 and GSE104471 both shared DNA regions methylation levels in monoallergen atopic asthmatic and healthy people. We discovered that these two groups obtained good classification either in t-SNE that can be seen directly (Figures 2(a) and 2(b)) or in the randomForest (the error rate: 0%) method. Then, a dataset on food allergies was found to test DNA regions' methylation levels among egg allergy patients, peanut allergy patients, and healthy people. We still observed a good classification at t-SNE (Figure 2(c)) and randomForest (Figure 2(d), the error rate: 0%). Therefore, we got a preliminary conclusion that there can be a perfect distinction between monoallergen atopic asthmatics with health and those with different allergen allergies after using machine learning analysis for a few specific DNA methylation regions.

**Figure 3:**
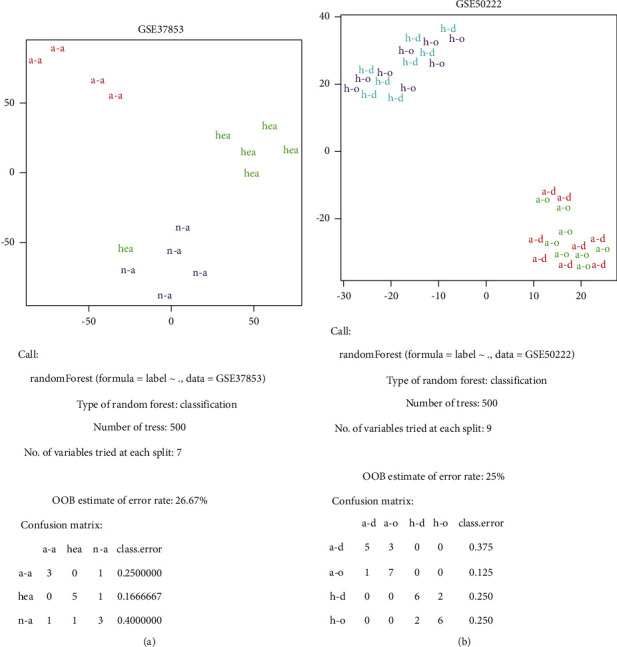
(a) The result of GSE37853 by t-SNE and randomForest. “a-a” represents atopic asthmatic patients, “n-a” represents nonatopic asthmatic patients, and “hea” represents healthy controls. (b) The result of GSE50222 by t-SNE and randomForest. “a-d” represents allergic patients during the pollen season, “a-o” represents allergic patients outside of the pollen season, “h-d” represents healthy people during the pollen season, and “h-o” represents healthy people outside of the pollen season.

The GSE37853 [38] data described the DNA methylation levels differently in atopic allergy patients, nonatopic allergy patients, and healthy people. The GSE50222 [39] data described how the DNA methylation level changes in allergic patients correlate with symptom severity, which followed DNA methylation levels outside and during the pollen season in healthy people and allergic patients. There are defective classifications exposed with a low error rate. Uncertain allergens and small samples available for “learning” may cause the GSE37852 data classification error (Figure 3(a)). In the GSE50222 data, allergy groups and healthy groups both recorded the same DNA methylation information at different seasons, which caused intragroup samples to become similar even if they were different and made a low error rate; however, allergic patients and healthy controls were still classified accurately (Figure 3(b)).

**Figure 4:**
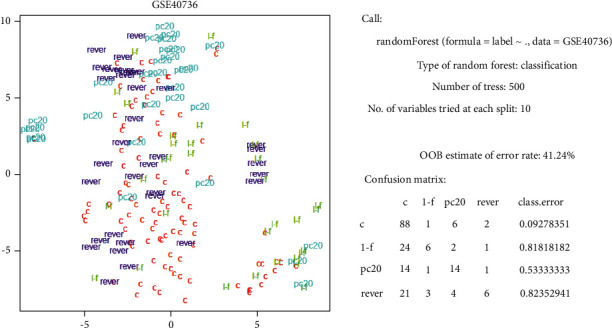
The result of GSE40736 by t-SNE and randomForest. “l-f” represents lung_function, “pc20” represents PC20, “rever” represents reversible, and “c” represents healthy control.

GSE40736 [40] data recorded allergy patients with several symptoms, and we tried to make classifications with the “Subtype” items (“non,” “lung_function,” “PC20,” and “reversible”) of this dataset. However, we failed to obtain slightly different DNA regions and had awful results (Figure 4).

### 2.2. Whole-Genome DNA Sequencing Results

The genome DNA sequencing data are viable after quality control (Table 1). Then, we analyzed the DNA regions (200 bp) methylation levels difference by Genome-Wide Association Studies (GWAS) and the single-nucleotide site methylation difference. The results were surprising. The GWAS result provided abundant different regions (*p* < 0.05) of annotated genes (*n* = 945), but the single-nucleotide methylation different sites had only few annotated genes (*n* = 466 of 2239), and these sites of annotated genes are always distant from the TSS. The far-TSS single-nucleotide methylation changes are always observed on dissociative single CpGs nearby some tandem repeats or intervening sequences. Also, these different single nucleotides are not detected in the different regions by GWAS.

## 3. Discussion

Though only few data were provided for computer-based “learning” and were not integrated (the data type: atypism; the primary data are not available), the preliminary conclusion obtained was that the specific allergen-related DNA methylation can be used to perform atopic allergen-allergy classification. However, the same method cannot discriminate the different anaphylactic symptoms. Moreover, the same method can determine whether allergic patients or healthy people or both were classified at different seasons. Therefore, we deduced that DNA methylation changed after exposure to atopic allergens, which was associated with specific allergens. These changes and anaphylactic symptoms both occurred after exposure to allergens, which may have caused the symptoms to not be classified.

In the whole process of GEO data analysis, we observed that there was a significant difference, but not high enough, and intragroup stability was low. It may be because the observed entities are DNA regions' methylation levels, which include more sequence information. In general, sequencing the genome nucleotide information, finding the difference DNA region, annotating genes or gene sites, analyzing the associated gene expression, exploring the possible regulatory mechanism, and obtaining a complete theory are a simple total process of the mechanisms associated with genetics of a disease [12, 41–44]. However, this pattern is less helpful for allergy diagnosis. But, allergen exposure can cause DNA methylation levels change, as observed in the GSE50222 data (Figure 3), and are classable. We intend to use cutting-off pilot processes by Occam's Razor, “Entities should not be multiplied unnecessarily (*Non sunt multiplicanda entia sine necessitate*),” which is the philosophy of idiographic machine learning formula selection, to find DNA nucleotides associated with methylation after allergen exposure.

Therefore, we devised and performed experiments on peripheral blood DNA methylation sequencing to compare single-nucleotide with DNA region methylated levels, which could help to find more suitable marks. We obtained almost the opposite result for the different typological methylated levels.

The sequencing results prompt that there are some single-nucleotide methylation changes and the most changes maybe concealed from the found different DNA regions, in the nonannotated gene regions between healthy people and allergy patients after allergen exposure, in spite of the fact that the simple numbers are too low. We conjectured that DNA methylation has timely changes that should exist when allergic constitution patients are exposed to atopic allergens, and DNA methylation could be distinguished from healthy people.

Therefore, we suggest conducting more “pragmatic” research to directly find special single-nucleotide changes after exposure to an atopic allergen instead of doing complex correlativity and trying to accurately diagnose anaphylaxis using DNA methylation marks to form a single CpG methylation-based classification by machine learning. The DNA methylation potential in the field of forensic medicine is great as an epigenetic mark in spite of too little research and application. DNA methylation also captures responses to environmental factors and changes with it [14, 39], so care is needed when applying this information to personal identification. Meanwhile, our analysis results of sequencing also remind us to pay more attention to single-nucleotide methylation.

## 4. Methods

### 4.1. Data Acquisition and Processing

Searching in the Gene Expression Omnibus (GEO, https://www.ncbi.nlm.nih.gov/geo/) with “allergy,” “DNA methylation,” and “*Homo sapiens*” as the key words and selecting the cell type as peripheral blood mononuclear cells (PBMCs), we got 6 valuable results (GSE59999 [37], GSE73745 [35], GSE104471 [36], GSE37853 [38], GSE50222 [39], and GSE40736 [40]). In these 6 studies, the other covariates such as gender, age, and ethnicity were controlled by every independent dataset as an independent study. We have developed the same criteria for analysis to evaluate feasibility: (a) the datasets were analyzed with GEO2R [45] with *p* < 0.05 as cutoff values and extracted the *β* value of TOP100 probes of each sample to one document (did not include censored data); (b) we used R package Rtsne (0.15) [46] to carry out t-distributed stochastic neighbor embedding (t-SNE), which is a nonlinear dimensionality reduction technique well suited for embedding high-dimensional data for visualization in a low-dimensional space of two or three dimensions by setting the parameters as dims = 2, perplexity = 30(the perplexity should less than [nrow(X) − 1]/3), and max_iter = 500, to estimate the samples data visualization; (c) the data were preprocessed using R package randomForest (4.6–14) [47] by setting the parameters as tree = 500 and the training set : test set = 7 : 3 randomization to find simple classification model; and (d) R package heat-map was used to perform heat-maps, and the clustering results were added in the plots.

### 4.2. Study Population

We recruited participants (*n* = 30) aged between 25 and 35 who had lived in Taiyuan, Shanxi Province, for a long time and had allergic symptoms and a positive skin prick test (SPT) from September to October (wormwood pollen season). Total IgE and sIgE were assayed by the AllergyScreen® test (Mediwiss Analytic GmbH, Moers, Germany) according to the manufacturer's instructions. All the participants (*n* = 30) were tested for 19 allergens, composed of 10 types of common aeroallergens and 9 types of food allergens. Among these allergens, the aeroallergens included house dust, pteronyssinus, short ragweed, estragon, mulberry, cat epithelium, dog epithelium, cockroach, mould mixture (*Penicillium notatum*, branch spore mildew, *Aspergillus fumigates,* and *Alternaria*), trees (mixture of cypress, elm, phoenix tree, *Betula*, *Fraxinus chinensis* Roxb, willow, and cottonwood), and grass (ragweed and wormwood). The food allergens included cow milk, beef, cashew-peanut-soybean, egg white/yolk, prawn, crab, cowry, mango-peach-apple-cherry, and pineapple. The sIgE level >0.35 IU/ml was considered positive, and the significant reference range of serum total IgE was defined as >100 IU/ml. Finally, only 3 participants were defined as wormwood monoallergen atopic rhinitis patients (the wormwood-specific IgE (sIgE) >17.5 IU/ml and total IgE >200 IU/ml but other common allergens sIgE all <0.35 IU/ml in peripheral blood serum). Thus, we selected the 3 wormwood monoallergen atopic rhinitis patients and 3 healthy people for methylation analysis.

### 4.3. Whole-Genome DNA Sequencing

Genomic DNA from peripheral blood mononuclear cell samples of wormwood monoallergen atopic rhinitis patients (*n* = 3) and healthy people (*n* = 3) during the pollen season was isolated using the DNA extraction kit (Omega, USA). We structured DNA libraries using the TruSeq Nano DNA LT Sample Prep Kit (Illumina, San Diego, CA, USA) and used bisulfite to convert all unmethylated cytosine (C) in genomic DNA into uracil (U) using EpiTect® Fast Bisulfite (Qiagen, Germany) (the bisulfite conversion rate: 99%). The DNA library quality control was performed by using the Agilent 2100 Bioanalyzer (Agilent Technologies, California). The whole-genome DNA methylation was sequenced by using a Whole-Genome Shotgun (WGS) [48] with Illumina HiSeq (Illumina, San Diego, CA, USA). After clearing the linker sequence and low-quality reads, Bismark (0.19.0) [49] was used to align the reads to the genome (GRCh38/gh38) through Bowtie2(2.2.3) [50]; then, we identified base transversion events, classified, and counted them.

### 4.4. Differential Methylation Analysis

RSeQC [51] (version 2.5; https://rseqc.sourceforge.net/) was used to count the distribution of methylation in different regions of genes. Differential DNA methylation regions (DMR) analysis between samples was performed on the R bioconductor package methylKit (1.19.0) [52]. Then, we annotated location information, the chromosome segment, and upstream and downstream information by contrasting with Ensembl 89 (https://asia.ensembl.org/index.html). Following, Gene Ontology (GO, https://geneontology.org/) and Kyoto Encyclopedia of Genes and Genomes (KEGG, https://www.genome.jp/kegg/) enrichment analysis of genes was conducted to get the DMR in the promoter regions. Finally, we obtained associated different annotated genes and DNA regions. In addition, we also used the methylKit to analyze the difference between the methylation of single-nucleotide sites and annotated location information but no GO and KEGG enrichment analysis.

The analysis of the data was performed in RStudio (Version 1.2.1335; https://www.rstudio.com/) using an R environment (version 3.6.0; https://www.R-project.org). All experiments were permitted by the Ethics Committee of Shanxi Medical University (2017LL073), and all methods were performed in accordance with the relevant guidelines and regulations.

## Figures and Tables

**Table 1:** The sequencing quality evaluation.

Sample	Reads num.	Total bases (bp)	GC (%)	Error <1 (%)	Error <0.1 (%)
a9	305,727,176	45,859,076,400	21.34	95.31	90.30
a10	333,038,672	49,955,800,800	21.37	95.28	90.36
a12	300,768,056	45,115,208,400	21.24	95.49	90.64
c1	297,128,974	44,569,346,100	21.19	95.35	90.34
c2	301,946,840	45,292,026,000	21.23	95.23	90.25
c3	337,787,768	50,668,165,200	21.28	95.48	90.63

Error: single-nucleotide distinguished error. The quality scores all bases of per sample in the attachment document.

## Data Availability

The datasets used and/or analyzed during the current study are available from the corresponding author on reasonable request.
